# A Chance for Attributable Agency

**DOI:** 10.1007/s11023-015-9381-y

**Published:** 2015-08-04

**Authors:** Hans J. Briegel, Thomas Müller

**Affiliations:** Institut für Theoretische Physik, Universität Innsbruck, Technikerstraße 25, 6020 Innsbruck, Austria; Institut für Quantenoptik und Quanteninformation der Österreichischen Akademie der Wissenschaften, Otto Hittmair-Platz 1, 6020 Innsbruck, Austria; Department of Philosophy, University of Konstanz, Fach 17, 78457 Konstanz, Germany

**Keywords:** Free will, Agency, Libertarianism, Indeterminism, Projective simulation

## Abstract

Can we sensibly attribute some of the happenings in our world to the agency of some of the things around us? We do this all the time, but there are conceptual challenges purporting to show that attributable agency, and specifically one of its most important subspecies, human free agency, is incoherent. We address these challenges in a novel way: rather than merely rebutting specific arguments, we discuss a concrete model that we claim positively illustrates attributable agency in an indeterministic setting. The model, recently introduced by one of the authors in the context of artificial intelligence, shows that an agent with a sufficiently complex memory organization can employ indeterministic happenings in a meaningful way. We claim that these considerations successfully counter arguments against the coherence of libertarian (indeterminism-based) free will.

## Introduction

We are part of a world that contains *agents*: things that react to environmental stimuli in flexible yet sensible ways, and to whose active powers we attribute many of the happenings around us. We ourselves, human beings, are prime examples of such agents, but we ascribe agency much more widely; in fact, to most if not all animals, and to some artifacts like robots that we made ourselves. The cat pushed the book off the table, Sue went to London, the bug hid under a leaf, my mobile phone told me to turn left, the dog ate my homework. These are ordinary ways of thinking and talking, and while there are certainly some borderline cases or reasons to correct some of our attributions of agency in the light of further considerations, it seems outrageous to assume that none of these attributions are warranted. Yet, the notion of attributable agency is conceptually problematic in a way that casts doubt on its coherence.^1^ Most of the discussion of these problems is directed at a specific case of attributable agency, human free will as the basis for the *moral* attribution of actions—but as we will show, the conceptual problems are much more general, and more basic than that. We lay out these problems and set them in a general context in Section “The Problem of Attributable Agency”. In Section “Projective Simulation: A Model for Attributable Agency”, we describe an indeterministic model, recently introduced by one of the authors in the context of artificial intelligence (Briegel 2012; Briegel and De las Cuevas 2012), that we claim successfully meets the conceptual challenges of attributable agency. We discuss this model and its implications in Section “Discussion”, and we draw some conclusions in Section “Conclusion”.

## The Problem of Attributable Agency

As we said, attributable agency—the agency of concrete agents such as animals—is all around us; how could that be problematic? The problem, generally speaking, comes from trying to understand an agent and its actions as part of the natural order of things that is the subject of scientific investigation. It is a problem, one could say, of bringing together the two views on our world that Sellars (1963) called the “manifest” and the “scientific image of man”, and as such, a core problem of philosophy.

### The Agency Dilemma

For concreteness, let us consider a would-be agent, $\alpha$ (some actually existing, persisting thing, e.g., the cat), and some actual past event, *e*, occurring at $t_e$ (such as the change of a property of a persisting thing, e.g., the book falling off the table). Under which conditions could it be warranted to attribute *e* to $\alpha$? (When would it make sense to say that the book’s fall is due to the cat?) We would normally take this question to concern specific details of the situation. (Normally, if the cat touched the book and it fell, the fall was due to the cat, but not if, e.g., you pushed her against the book.) But the following dilemma seems to show that such attribution can *never* be warranted. That dilemma’s usual habitat is the free will debate, and we will draw some connections to that debate and its well-entrenched terminology, but the dilemma applies much more widely, and in fact threatens to undermine not just the complex and controversial notion of human free will, but what is, quite uncontroversially, one of its necessary preconditions: attributable agency in general.

The two horns of our dilemma follow a major watershed in metaphysics. Given the actual situation *s* at some time $t_s$ before the occurrence of *e*, it is either the case that (a) the only possibility for $t_e$ was that *e* occurs, or (b) it was possible that at $t_e$, *e* does not occur. More generally, either (a) the world is deterministic, such that at any time, there is exactly one possible future course of events, or (b) the world is indeterministic, i.e., not deterministic: at some times, there is more than one possibility for what will happen in the future.^2^

Consider the deterministic horn first: does attributable agency make sense under the assumption of determinism? This is an extremely controversial subject, on which much ink has been spilt. So-called *compatibilists* with respect to free will have devised various ways of making sense of the idea: even when facing a deterministic toy world (such as the world of Conway’s game of Life that figures prominently in Dennett (2003)), we can adopt a stance according to which we can describe what goes on in terms of attributable agency. On the other hand, there is the powerful intuition that if in the real world, given *s* at $t_s$ (which can be the situation thousands of years before our would-be agent $\alpha$ is born), it is fixed that *e* will occur at $t_e$, then $\alpha$ can’t really have anything decisive to do with *e*, and any attribution of agency will have to be by courtesy only. In fact, in the deterministic setting, it seems hard to ascribe to $\alpha$ an identity as an agent. Of course, $\alpha$’s body is causally involved in what happens, like a cogwheel in a machine, but $\alpha$ makes no real difference to what is going on: *e* is not due to the agent, and thus cannot be attributed to $\alpha$. This strong intuition has been formalized as the so-called “consequence argument” by Van Inwagen (1983), who glosses his argument as follows:

If determinism is true, then our acts are the consequences of the laws of nature and events in the remote past. But it is not up to us what went on before we were born, and neither is it up to us what the laws of nature are. Therefore, the consequences of those things (including our present acts) are not up to us. (Van Inwagen 1983, v)

This argument is considered a major problem for compatibilism, because it appears to rule out the possibility of attributable agency under determinism. It is not the purpose of this paper to refute compatibilism, and so we need not enter a lengthy discussion of the consequence argument. The purpose of our paper is to argue for the coherence of libertarianism, or rather, to show that there can be attributable agency under indeterminism. Let us just state, for the record, that we have not yet seen a successful rebuttal of the consequence argument in the literature. At any rate, considering the deterministic option offers no help for an understanding of attributable agency in our concrete world, because we have excellent reasons—the best scientific reasons we can have for any metaphysical belief, in fact—to believe that our world is deeply indeterministic. Thus, even if a compatibilist notion of attributable agency could be defended, that notion would not pertain to our world, and would thereby be useless.^3^

Since the issue is so controversial, let us add three remarks. First, compatibilism is, strictly speaking, just concerned with the logical question of the compatibility of determinism and attributable agency (or free will), and an answer to that question can give insights into the notion of agency even if determinism is false. Furthermore, many compatibilists are avowedly science-friendly (“naturalists”), and are interested in how an acknowledgment of quantum indeterminism affects a compatibilist defense of free will. Reactions to this are divided. Some compatibilists explicitly proclaim to be agnostic as to the determinism or indeterminism of our world.^4^ Such compatibilists, however, often go on to focus on the deterministic option only, ignoring the challenge to develop a sensible notion of attributable agency under indeterminism. This strategy relies on the idea that whatever randomness there is in the world, it will only be a small disturbance on top of an otherwise deterministic course of events that is all that really matters. But this is fallacious. It is true that indeterminism can occur in such a way that all that surfaces is a small, insignificant fluctuation around a stable and, for all practical purposes, deterministic statistical average. Consider a laser beam: its individual photons are produced by stimulated emission, which is an indeterministic quantum mechanical process. Due to the large number of photons, however, a stable average intensity is achieved, and for most applications, fluctuations can be ignored. But not all indeterminism is like that. The very fact that we know about quantum indeterminism implies, of course, that it can have macroscopic effects that can be detected by us. A significant milestone here is the single electron version of the double slit experiment (Jönsson 1961), which was voted the world’s most beautiful experiment in 2002 by *Physics World*. More recent experiments demonstrated explicitly the stochastic build-up of an interference pattern through single-particle detection events at random positions on a screen (Tonomura et al. 1989; Juffmann et al. 2009). For references outside of a laboratory context, consider the fact that some online casinos advertise their use of quantum randomness as a security feature. In such a case, the effect of a photon being transmitted or deflected by a beam splitter will influence the hand you are dealt, which can have a significant impact on your bank account. In brief, quantum randomness can make a macroscopic difference that we can perceive, contrary to what many compatibilist participants in the agency debate assume.^5^ A true agnostic, who allows for concrete actions of an agent to be undetermined, has to face a double task: to develop a sensible notion of attributable agency under determinism, and to develop such a notion under significant indeterminism. In so far as we proclaim to take up the latter task only, our aim is in fact strictly narrower than that of the serious agnostic. You can’t be an agnostic compatibilist and blame the libertarians for not having done their job. If you blame the libertarian, this shows that your agnosticism is just a variant of deterministic compatibilism that allows only for insignificant, undetectable random noise, and as such, is empirically refuted. Second, other compatibilists argue that no matter quantum indeterminism, *at the level of the brain*, determinism reigns. It is true that individual neurons do not show the indeterministic behavior of a photon hitting a beam splitter—they show a fairly regular behavior. But it is fallacious to argue from this to determinism of the brain. Never mind the stochastic behavior of individual neurons—the brain is an open system, and indeterminism in the environment can make a perceptible and also a behavioral difference to an agent, as pointed out above (think, e.g., also of hearing a Geiger counter click). Third, it appears that many compatibilists, when pressed, do agree that we are initially inclined to want a notion of attributable agency that is connected with an open future and thus, with indeterminism. A common reaction on the compatibilist side is, however, to argue that *since no sensible notion of attributable agency—or freedom—under indeterminism seems to be forthcoming*, the compatibilist option does deliver all the freedom we can have and, thus, all the freedom “worth wanting” (Dennett 1984). If you can’t have what you love, love what you have: a sensible maxim for us finite human beings indeed. As we will try to show, however, indeterminism can deliver a more sensible notion of attributable agency than commonly supposed.

So, is the grass all green on the indeterminist’s side of the fence? No, it isn’t, and in fact the conceptual problems of attributable agency under indeterminism are seen by many philosophers to be insurmountable. This brings us to the second horn of the dilemma. In a nutshell, the problem is that if given *s* at $t_s$, the occurrence of *e* at $t_e$ isn’t determined, then it seems *e* must be a chance happening—and what is due to chance, isn’t due to the agent. This challenge is presented in a number of different forms, and has recently been the focus of increased attention.^6^ In one form, explicitly directed at human intentional agency as involving (perhaps conscious) reasons for acting, the challenge is known as the “luck objection”: if $\alpha$ brings about *e*, but could have brought about *f* instead, then indeterminism means that there can have been no sufficient reason for $\alpha$ to choose *e* instead of *f*. Thus, unless $\alpha$ meant to let itself be directed by randomness, e.g., as a means for tie-breaking,^7^ this means that the occurrence of *e* cannot be attributed to $\alpha$ after all: it seems that $\alpha$ should have, but lacks, a contrastive reasons-explanation for the occurrence of *e*.^8^ This objection is interesting, but its discussion leads to the topic of *consciously acting for reasons*, which is a phenomenon that, like human free will (if indeed it is different from free will at all), is vastly more complex than the notion of attributable agency that we wish to tackle in this paper. At any rate, attributable agency is surely a necessary precondition for higher forms of agency, and so, any conceptual trouble for attributable agency is *a fortiori* a problem for the notion of consciously acting for a reason as well. At the level of mere attributable agency, the challenge is perhaps best expressed by what, following Van Inwagen (2000), is known as the “replay argument”. Assume that actually, starting with the concrete situation *s* at time $t_s$ (which we may take to be pretty close to $t_e$), *e* occurred at $t_e$, and we wish to attribute *e*’s happening to $\alpha$. Now assume that we replay the course of events starting with *s* a number of times—this is of course something we cannot do, but maybe God could do it, and at least we seem to be able to imagine it coherently. By assumption, the occurrence of *e* was not determined, so that among the *n* runs in total, there will be a number $n^+$ of runs in which *e* occurs, and $n^-$ runs in which *e* fails to occur at $t_e$. Thus, there is an objective, ground-floor probability, roughly equal to $n^+/n$, for *e* to occur given *s*. But, so the argument goes, this shows that the occurrence of *e* is just a chance happening, and as such, not due to the agent.

On both horns of the dilemma, it seems, the notion of attributable agency cannot find a sufficient foothold. In any case in which it makes sense to posit a self-identical agent at all, such an agent would either be enslaved by a deterministic course of events that started long before it was born, or it would be enslaved by random happenings that toss the agent to and fro on an erratic course. Neither scenario warrants the label “attributable agency”. Furthermore, the two horns form an exhaustive disjunction: either the world is fundamentally deterministic, or it isn’t, there cannot be a third way. So attributable agency, it seems, makes no sense. But this is outrageous. Isn’t there a way out?

### Indeterminism: Friend or Foe?

As we said, we see no useful role for a notion of attributable agency under determinism: even if such a notion turns out to be tenable, it will not help us to understand attributable agency in our world, which we know to be indeterministic.^9^ So, we need to look more closely at the prospects of understanding attributable agency under indeterminism.^10^

In many discussions of agency, it is acknowledged that objective indeterminism opens up a new range of ontological possibilities when compared with determinism. It is a simple conceptual fact that under indeterminism, the future is open, while under determinism, it isn’t. In an indeterministic world, more than one thing can happen. As the replay argument shows, there is however also reason for skepticism as to the consequences that indeterminism has for agency and control. Can there even be a stable, let alone a reliable agent in an indeterministic world?

This question takes indeterminism as a threat for reliability. And surely there is some truth to that. Put bluntly, how can there even be reliable mechanisms in an indeterministic world? Well, our world is indeterministic, and it contains reliable mechanisms. Biological cells are perfect examples of the successful harnessing of indeterminism in the interest of a functioning whole. The building blocks for protein synthesis move randomly through a cell’s interior, but protein synthesis is a very efficient process. Ion channels in a cell’s membrane behave stochastically, but the polarization or depolarization of cells is an efficient means of signal transmission. These are examples—there are many more—of the successful (even though not exceptionless) harnessing of indeterminism, of counteracting an erratic micro-dynamics at some higher level of functional organization.

Is that all that we can say about indeterminism: it is there, but it can be overcome by error correction—by clever design in the interest of deterministic mechanisms? That would stress a generally negative outlook on indeterminism, and not suggest that indeterminism can play any *useful* role in agency. Maybe it is what nature has to deal with, fundamentally, but luckily it can be corrected for?

In fact, at higher levels of organization indeterminism can play out some real strengths. An animal can have a competitive edge over a predator if it does not behave fully predictably, but rather has some random element in its avoidance behavior.^11^ Such truly erratic behavior is most probably not implemented by *simulated* randomness—building a deterministic mechanism that behaves almost indeterministically, like a computer’s pseudo-random-number generator—but by the *real* randomness in nature that is present as a raw resource.^12^

Is there a useful role for indeterminism at the higher level of attributable agency, apart from the purposive use of randomness for tie-breaking? We already said that attributing the *use* of randomness to an agent seems quite uncontroversial. But can we also attribute to it an individual event that is the concrete outcome of a random process? Is that ever warranted? Or is randomness just a threat at that level, without a positive role?

Much here depends on the underlying assumptions about understandable, attributable agency. A standard model of rational agency (a subcase of attributable agency) looks as follows:^13^ based on its beliefs about the world, an agent generates a number of options for action, by some computational process. These options are then weighed, e.g., by their expected payoff according to some metric that takes into consideration the agent’s desires or preferences, yielding (at least according to many models) values that are pairwise comparable (e.g., real-valued expected utilities). Then the best option is selected and executed. This is a deterministic algorithm. The only role that randomness can usefully play, except perhaps at the first stage (where, e.g., some random sampling of the space of options may be necessary if the agent would otherwise create too many options), is for tie-breaking in the case in which two or more options are assigned the same weight (or, assuming only partially ordered weights, when different maximal weights are incomparable). Any other use of randomness in option-selection on this model will lead to suboptimal performance, since sometimes, options will be selected for which the agent has a strictly better alternative. How could that help rationality?

Well, if that is what rationality is, then it couldn’t; but it isn’t. The agent model just described is certainly useful for some applications, and can lead to sensible results. But it is quite implausible to assume that we are like that, and the model we are proposing is not of this kind either.

## Projective Simulation: A Model for Attributable Agency

In this section, we will introduce the model of projective simulation that uses indeterminism as a resource in such a way that, as we claim, the concrete outcome of a random process *can* be sensibly attributed to an agent.

### Randomness as a Resource

Randomness is a much-wanted resource in modern society and technology. It is e.g. used in computer simulations of various processes in biology, ecology and economy, and it also forms the basis of modern data encryption and communication systems.

The question, to what extent the sources underlying these applications can be certified as truly random, is the subject of considerable attention and of high practical relevance. For example, while so-called pseudo-random number generators may be sufficient for many applications, they are considered (and in several cases actually proved to be) insecure for cryptographic applications that need to guarantee the privacy and security of a communication channel.^14^ On the other hand, truly random events can be generated by exploiting fundamental physical processes such as the radioactive decay of a nucleus or the passage of single photons through a beam splitter. Quantum random number generators based on similar processes have been developed and are being used today.^15^

The fact that such applications exist shows that some of the empirical agents (i.e., humans), whose nature is the topic of our investigation, are not only exposed to randomness in their daily life, but they even exploit it as part of their agency. This implies that even a compatibilist approach (advocating a deterministic notion of agency) has to account for empirical agents that integrate random events into their agency, not only without compromising their rational behavior, but as the very result of their rational reasoning.

The question is thus not whether there is any room for randomness in our model of the world. The task is to show, on the positive side, how randomness can be included into a model of a rational agent itself, and what role it plays regarding the possibility of freedom and attributable agency.

### Projective Simulation

From the phenomenology of our own agency, we are familiar with random distractions in our daily business, be it practical, like having to answer the phone when we are preparing a meal, or theoretical, when we are thinking about a problem.

Mental work has an interesting phenomenology of its own. In particular, creative mental work, like writing a novel, composing music, or struggling with a mathematical proof, is usually plagued with many distractions. Interestingly, many of these distractions are self-generated and seem to occur without a particular reason, other than a lack of concentration or focus in following our business. We ourselves are familiar with falling into chains of thoughts like the following:

While remembering the recent birthday party of our son, it returns to my mind that one of his friends did not come because he had an accident with the bike and broke his leg ... both of his parents work at Wall Street and I am wondering how the family will cope with the financial crisis ... who knows how all of this will affect our pensions and savings ... scenes from my childhood pop into my mind, from the days when we used to visit my grandma in Hungary, every year before she suddenly passed away, this was a wonderful time ... I don’t know what our health care will be like when I am going to retire ... it was very cute when all of the kids on the birthday party suddenly formed a circle and started singing happy birthday ...

Such apparent “stumbling” through our memories happens not only when we try to remember things and let our mind drift about, but similar observations can be made when we contemplate and try to solve a scientific problem. The main difference lies in the context and the “space” of associated thoughts, but the phenomenology is similar. Obviously what we are describing is nothing but “association” and the capability of our mind to relate “similar things”. Such association can be rambling and almost accidental, as if our mind were, at times, to enter into a state of random motion. Usually we would interpret such random motion as a surface phenomenon, ultimately caused by hidden deterministic processes (possibly unconscious) or by some external distraction.

But what if such a random walk through our memories was not the result of some external distraction or of lack of focus? What if it was a constitutive element of our remembering and thinking?

Random stumbling through memory can indeed have a positive effect. It allows us to break out of a “line of thought” that may lead to a dead end or whose end is not well-defined to begin with (like in genuine research). Clearly, such random associations can only be constructive if they are not too violent and don’t destroy the overall coherence of our agency—that is, if at the end there will be a good novel or a finished proof, rather than an overall state of confusion.

A formal model of agency for which randomness plays indeed a constructive role has been described in Briegel and De las Cuevas (2012). The model is formulated within the conceptual framework of “artificial agents” (Russell and Norvig 2011), which is a modern platform for the study of artificial intelligence. An artificial agent is equipped with sensors and actuators, with which it can perceive signals from its environment and act on it. Specific types of agents are usually defined by their “internal program” which relates perceptual input to resulting actions. The agent model introduced in Briegel and De las Cuevas (2012) is based on “projective simulation” (PS), which can be seen as a novel scheme of deliberation for agents that is not based on symbolic information processing. In the following, we summarize the essential elements of PS; for a detailed treatment, we refer the reader to the original papers.

**Fig. 1:**
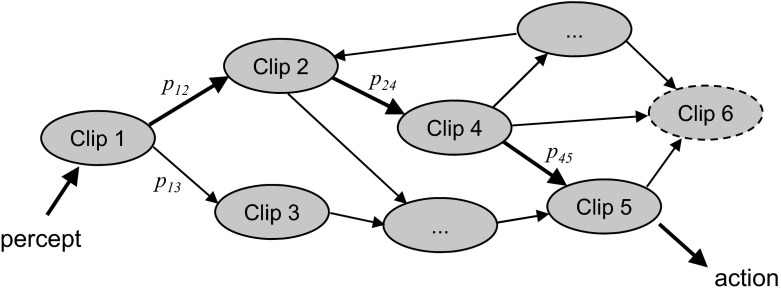
Model of episodic and compositional memory (ECM) as a stochastic network of clips. Perceptual input, indicated by the *bold arrow on the left*, triggers a random motion through memory. The random motion between different clips is governed by transition weights (probabilities) that are continuously modified under the agent’s experience. $p_{ij}$ denotes the probability of transition from Clip *i* to Clip *j*. If a sequence of transitions is taken that leads to a rewarded action (here illustrated by the sequence 1-2-4-5), the weights of all transitions involved in that sequence will be increased. Clips can also be modified and newly created (here indicated as Clip 6 with *dashed boundary*) as part of the learning process. (Figure adapted from Briegel and De las Cuevas (2012).)

PS is based on a close interplay between the actions of the agent and a specific type of memory, called episodic and compositional memory (ECM), whose primary function is to store and reinvoke past experience. ECM is organized as a network of so-called “clips”, which are the basic units of memory (see Fig. 1). They correspond to fragments of episodic memory, reminiscent of very short sequences of a movie, or a melody. A call of ECM, triggered by some perceptual input, leads to a random motion through the space (network) of clips.

An illustration is given in Fig. 1, where perceptual input excites some specific clip (labeled Clip 1) in the memory. The excitation of Clip 1 will then propagate and lead, with some probability $p_{1j}$, to the excitation of a neighboring clip *j* in the network. As the process continues, it will generate a sequence of transitions between different clips, which corresponds to a recall and random reassembly of episodic fragments from the agent’s past. The process stops when an excited clip couples out of memory and triggers motor action.

The random motion between different clips is governed by weights (transition probabilities) that are continuously modified under the agent’s experience. This can be implemented, for example, by Bayesian updating within a reinforcement scheme. If an action is rewarded, the weights of all transitions (for example 1-2-4-5 in Fig. 1) that led from the percept to that action will be increased. The weights of the entire clip network thereby change over time, and they thus reflect the agent’s learning history.

Central to the concept of the episodic-compositional memory is the possibility that clips may themselves be randomly created and modified as part of the simulation process (see Fig. 1). Random clip sequences which are thereby generated will introduce new, “fictitious” episodes that may never have happened in the agent’s past. Regardless of the fictitious character of such episodes, however, they can trigger factual action by the same mechanism (e.g., screening and feature detection) as the recalling of “real” episodes.

With the ECM as described, the agent is endowed with a simulation platform to “play with” its past experience, without leading to immediate motor action. By the process of PS, the agent, triggered by perceptual input, is continuously recalling episodic memory, thereby reshuffling, mutating, and creating new episodic fragments in the process. This is effectively used by the agent to simulate conceivable future experience on the basis of past experience, whereby the notion and range of the *conceivable* is defined by the rules of clip composition and variation (Briegel 2012).

Remarkably, all described transitions within ECM that constitute the PS are assumed to be random, following only certain probability distributions. Random processes drive both the transitions between existing clips and the creation of new clips. The model of PS is thus an intrinsically indeterministic model that uses random processes as part of its design.

In contrast to the discussion in Section “Randomness as a Resource”, randomness is here not something “external”, which the agent is confronted with and which it may need to correct or otherwise “deal with”. Instead, it is a constitutive part of the agent’s identity, which changes as the agent learns. This is an important point for our discussion of attributable agency. Crucially, the ECM provides a separate level and a “playground”, which is detached from the primary sensory and motor level. Given this separate level, randomness can now be exploited in a constructive way: whereas randomness that acts on the primary (e.g. motor) level leads to manifest erratic behavior, it becomes the driving force that creates (a primordial notion of) *options*^16^ when acting on the memory level (Briegel 2012).

It should be noted that for the phenomenology of behavior of the agent (e.g., regarding its learning efficiency), the ultimate nature of the random processes that generate the clip transitions (as described by the probabilities $p_{ij}$ in Fig. 1) is not important. If these probabilities are not generated by genuinely indeterministic processes (as in quantum physics) but, say, by some pseudo-random number generator, the agent will show similar behavior in most situations.^17^ In this sense, we have something to offer to the deterministic compatibilist as well: in a deterministic world (a world unlike ours), it may be possible to make sense of attributable agency if an agent contains reliable sources of apparent randomness. The main point is that at the level of functional organization that explains the dynamics of ECM in the model, the $p_{ij}$ clip-to-clip transition probabilities are “ground floor” probabilities that are not analyzed any further. In fact, assuming that pseudo-randomness is used (as in simulations of ECM on a standard computer), the added deterministic layer does not enhance our understanding of what the agent does—rather, such a deterministic background would be only accidentally related to the agent’s dynamical development, while the indeterministic random walk through clip space is constitutive of the agent’s development, and in this sense, not accidental. Note that via the model of PS, we have turned the table against the deterministic compatibilist. It is not that we model an agent as a deterministic system that can probably tolerate some randomness—rather, we model an agent as an indeterministic system that can probably tolerate some determinism. It is therefore clear that indeterminism is not a threat to the model, but a resource for attributable agency.

## Discussion

Having introduced the model of PS in Section “Projective Simulation: A Model for Attributable Agency”, what can we say about the conceptual problems of attribution in an indeterministic setting laid out in Section “The Problem of Attributable Agency”? Does the model succeed in laying the worries to rest? The model arguably has a good chance of capturing the dynamical nature of human action selection—but can it counter the charge of erratic randomness for single outcomes of that process?

### On the Indeterminism of Projective Simulation

The model is thoroughly indeterministic. The agent’s action, following some sensory input, comes about through a process that is doubly stochastic, involving both a random walk through the agent’s memory *and* the stochastically driven addition of additional “virtual” memory episodes. If an agent implementing the model were to face the same situation again, such as assumed in the replay argument (same perceptual input while being in the same internal state), its action could be different from the action it in fact showed—but it could also be the same. Also, depending on the concrete state of the agent’s memory, the set of actions that could possibly ensue given that situation, can be limited to a small subset of the agent’s full behavioral repertoire (even just a single option, as discussed in note 9), or it can, in the other limiting case, be *any* action in that repertoire.

The agent’s memory organization will for all practical purposes guarantee that the agent will never be in the same situation twice: its internal state keeps track of its history. This makes it sensible to think of the agent’s identity as developing historically, and that identity plays a crucial (yet not deterministic) role in its behavior when facing a certain situation.

According to the model of PS, an agent is not “small” in the sense criticized vehemently by, e.g., Dennett (2003), who notes that many discussions of agency treat an agent as a mere point and then wonder how an agent thus pictured cannot make a difference in the world. The model describes the agent’s internal organization at a level of detail that, while only referring to simple physical vocabulary, makes understandable what happens when the agent acts. But the agent is, in its constitution, not deterministic, but deeply indeterministic. That indeterminism is naturalistically motivated and is used *as a resource* that makes a degree of flexibility possible that seems implausible, if not impossible, to achieve in a deterministic setting.

### Revisiting the Replay Argument

In order to see whether the model can counter the charge of erratic randomness, let us confront it with the replay argument. The challenge, as described in Section “The Agency Dilemma”, is the following. Consider the real situation *s* at time $t_s<t_e$, and a real event *e* at $t_e$ that we wish to attribute to our agent $\alpha$. We assume that the agent is suitably described by the model of PS. Since that model is indeterministic, the situation *s* at $t_s$ does not normally determine or guarantee the occurrence of *e* at $t_e$.^18^ Now, consider a large number *n* of hypothetical replays starting with *s* at $t_s$. In a number $n^+$ of these, *e* occurs; in $n^-=n-n^+$ of the runs, *e* fails to occur. The rhetorics of the replay argument urges us to view the *actual* transition from $t_s$ to $t_e$ as a chance event whose objective chance of leading to event *e* we can estimate to be $n^+/n$. If *that* is what happened—a chance process occurred and forced the agent into one of its possible outcomes—then the agent had no role in it, the agent didn’t make a difference to what happens, and attribution is impossible.^19^ A story to the effect that it may be *evolutionarily beneficial* for the agent to let itself be enslaved by such chance happenings in certain situations (e.g., when determining the direction in which to flee from a predator, or in tie-breaking), may ground some weak notion of attribution, but what we can attribute there is not the concrete outcome (*e* happening), but only the general fact that a chancy happening occurs that involves the agent’s body and which plays some functional role for the agent.

This is not what we are looking for, and not enough to lay the replay argument to rest. What is demanded to meet that challenge, is an attribution of the *actual* outcome, in the *actual* course of events, in which indeterminism was not employed for tie-breaking. How can it be that the actual occurrence of the undetermined event *e* is *due to the agent*? Does projective simulation really change the game in that respect?

We claim that it does. The redescription of what happened as a single chance happening between times $t_s$ and $t_e$ in some sense amounts to deleting the agent from the story. But the model of PS gives a detailed account of the process going on within the agent that actually led from *s* to *e*: the agent is not a mere point, a mere label that we want to attach to the chance transition, but an extended being within which a chance process consisting of many steps is going on. This process affords *understanding* of the transition from *s* to *e*. Each of the steps in the actual random walk occurring in the agent’s memory that actually led to the occurrence of *e*, is individually a happening that plays a functional role for the agent as a persisting being. While these steps are *undetermined*, their occurrence is *not an accident*.

If we want to posit the question of attributability fairly, as an open question that could at least in principle receive a positive answer in some cases, we cannot drop the agent, its history and its identity from the picture. The process going on within the agent adds to that agent’s identity, it builds forth on the agent’s history in a sensible way. If we describe the happening that actually led to *e*’s occurrence within the model of projective simulation, we have to agree that we cannot account for the *individual* chance processes happening as the individual steps in the random walk through the agent’s memory space—we cannot offer a contrastive explanation why *this* step, rather than *that* one, took place.^20^ Each of these is what it is: a random event. But each of them also happens within a larger environment that fixes its role in the agent’s developing history.

As we said in the description of the model, an agent engaged in PS builds forth on its identity (its dynamically expanding and constantly updated memory). It has a history, which explains its current behavioral dispositions, which in the model are reflected by the internal transition probabilities between its memory-clips. These probabilities, which guide the random walk through clip space that leads to a concrete but undetermined action of the agent, are *not an accident*—they are the concrete trace of the agent’s history, of what it did and what that resulted in. And similarly, the individual steps in the random walk are *not an accident* either: they are the situated results of the agent’s historical development.

But isn’t there still a problem? What is wrong with the one-step description? In the model, each of the admittedly many steps leading from *s* to *e* has a concrete transition probability attached to it. Can’t we simply unravel the whole historical development, multiplying probabilities along the way (assuming, in the simplest case, a Markovian dynamics for the clip-to-clip transitions) and adding alternative paths that lead to *e*? Doesn’t this fix an objective probability for the occurrence of *e* given *s*, just as the replay argument claims? And doesn’t this show that we are thereby warranted in giving the agent-less description of what happens that shows that attributability is just a fluke, a manner of speaking that is ultimately not warranted?

In the simplest version of the agent, in which there is a discrete succession of percepts (input) and actions (output), it is true that we can give an “effective representation” of the agent’s behavioral disposition as a matrix of transition probabilities from the possible inputs to the possible outputs. At that level of coarse-graining, it is true that the PS agent’s mapping of inputs to outputs is a random process with fixed probabilities. But these probabilities are themselves the result of the agent’s (learning) history. Typically, under compelling circumstances, the agent will almost certainly do what is necessary (in the setting of reinforcement learning: choose the unique action that is highly rewarded/not punished), while in situations where its actions make no crucial difference (constant reward), the agent’s choice may indeed remain random. It is important to realize that this possibility does not affect the integrity of the agent.^21^ In summary, we can typically expect an agent, after some feedback from interactions with its environment, to have built up some rather fixed patterns of reaction to some stimuli. But such behavior does not have to be deterministic. We mentioned that indeterminism can have an advantage already for very simple scenarios. We may assume that flexibility is generally beneficial. What reason do we have for not attributing the concrete reaction of a flexible agent to a concrete percept, to the agent itself? The agent is not enslaved by some dubious external source of randomness leading to erratic behavior—rather, it shows flexible behavior of a form that can be explained, and understood, in terms of its past actions and experiences. The randomness inherent in the process does not change this—and in fact, the result of the action chosen according to the agent’s current memory state will lead to further development of the agent’s identity.

This is one component of our rebuttal of the charge of randomness: it makes sense. A second component comes from looking at *what is really happening* when the agent acts. It is true that in the simplest cases, we can give a summary description of the agent’s effective input–output behavior in terms of a matrix of transition probabilities. But this is not the level of the actually occurring processes. It is not the case that, parallel to the case of the BDI agent described above, the agent somehow computes its effective transition probabilities and then tosses a coin, or the appropriate number of coins, in order to determine an action to be executed. That would be an agent that subjects itself to an outside source of randomness, and we can agree that there is something fishy about attributing *the outcome of the coin-toss* to the agent. Such use of *external* randomness seems only to be understandable as due to the agent if it is used for simple tie-breaking (we *may* flip a coin to tell Buridan’s ass which haystack to choose)—but that is not our situation. What is really going on, at the level of dynamics that affords an understanding of the agent’s actions as meaningful and non-accidental, is different. That level is specified explicitly as the level of clip-to-clip transitions in the ECM model, independently of any computational implementation of the model, and is supported by phenomenological considerations of the dynamics of association above. The matrix of input-to-output transition probabilities, on the other hand, describes the agent from an external perspective that leaves out the crucial dynamics of the agent’s use of its memory (and thus, its history). The internal memory dynamics is stochastic as well, to be sure: a random walk through clip space. Each single random transition from one clip to another, however, is understandable from the internal perspective of the agent’s history. That level of description does justice to the actually occurring, objectively random processes of clip-to-clip transition. There is simply no random process, really, that leads from a percept to an action in one direct leap. Focusing on the real dynamics furthermore brings the dynamical nature of the agent’s memory back into view. As we said in describing the model, the set of clips in the agent’s memory isn’t fixed—new clips can be added via variations of existing memory content. In that way, the agent adds “conceivable happenings” to its simulation repertoire (“what else could have happened?”). This leads to the expansion of the agent’s range of possibilities. The summary description hides this element of the model, which is however crucial to the agent’s creativity, i.e., its capability of creating—by itself—new options for its subsequent actions.

A third component in rebutting the charge of erratic randomness, comes from taking seriously the fact that the agent is an open system that is in continuous interaction with its environment. Taking this aspect seriously leads us beyond the simplest model of PS, and it will lead one to reject the very setting within which the replay argument can arise. Since that argument plays such an important role, however, we do not want to rely exclusively on a move that rejects the argument out of hand. But we do believe that a serious consideration of the open systems nature of agents yields important insights and strong arguments in favor of indeterminism-based agency.

With respect to the idea of integrating the agent’s internal dynamics and providing an input–output transition probability matrix, the consequences of the status of the agent as an open system are as follows. In the actual course of events, which led to *e*, we can take the actual situation at each concrete simulation step into account, and the model indeed assigns objective probabilities to these *situated* steps. There is no guarantee, however, that we can assign meaningful transition probabilities in a hypothetical replay *unless the whole state of the universe is described in addition*. Maybe in one such replay a cosmic ray hits the agent and alters the transition probabilities. In an indeterministic world—the world we live in—the fact that no such ray hit the agent, gives us no reason to exclude it from a hypothetical replay. Unless, that is, we mean by a replay, a mere *repetition* of what actually happened. But that will be uninteresting, and will not give any support to the replay argument as an argument against attribution.

## Conclusion

In this paper we discussed a necessary precondition of human free will, which plays a role in animal agency as well: the notion of attributable agency. We showed that even this—rather simple—notion is subject to a dilemma that threatens its coherence: it seems that we cannot attribute agency under determinism (the agent cannot *make a difference* to what happens, as that was fixed long before its birth), nor under indeterminism (*the agent* cannot make a difference to what happens, since that is due to chance). We claim that putting the issue in these rather simple and non-committal terms, avoiding issues of practical wisdom and morality, is also helpful for the broader free will discussion, as it avoids running together considerations at different levels.

In a next step, we discussed the notion of indeterminism. While indeterminism is often seen as a threat to stable behavior, we showed that in many instances, indeterminism can be your friend. The image of indeterminism as pure erratic chance does not correspond to what we know about nature’s indeterminism (as exhibited, e.g., at the quantum level): natural indeterminism is not a matter of “anything goes”, but the random actualization of one of a limited number of determinate possibilities. The threat of randomness is therefore often misguided. Still this does not yet amount to a positive model of attributable agency under indeterminism.

In Section “Projective Simulation: A Model for Attributable Agency”, we described a model that, as we claim, succeeds at this positive task and describes attributable agency under indeterminism. The model, which originates in AI research by one of the authors (Briegel 2012; Briegel and De las Cuevas 2012), is built around a specific memory architecture that the agent employs in an associative fashion in order to “stumble”, via a random walk, from a given percept to some action. While the whole process is thoroughly stochastic, and no contrastive explanations of the individual steps as against alternative steps are available, the whole process can be understood as due to the agent. Crucially, this is not just because we may choose to adopt a certain stance towards the agent such that agency vocabulary becomes appropriate, but because what is happening, is part of the agent’s developing identity. The randomness driving the model is not an outside threat to the agent, but a constitutive, functionally central element of its organization.

We discussed the model of PS and its interpretation in Section “Discussion”, revisiting the replay argument against indeterminism-based attributable agency in detail. We claim that by presenting the model, we are able to successfully rebut the replay argument. We gave three main reasons: (1) The PS agent is not point-like. Our model treats an agent as a real thing with internal structure—its ECM—and not as a mere point-like label on a transition process. A replay must be allowed to affect the agent’s identity. (2) The PS agent acts rationally. The agent’s behavior, while stochastically driven, is not erratic, but makes sense. The agent’s ECM, encoding its past learning history and self-generated behavioral options and patterns, gives rise to agency that can be understood and attributed at the level of individual actions. (3) Finally, we remarked that the notion of a replay is not well-defined for real agents, who are open systems continuously interacting with their environments. Note that we have not just voiced counterarguments, but provided a positive instance of that which the replay argument suggests cannot exist. This shows that the argument is unsuccessful.

If we are right, we have shown *that* attributable agency is possible in a fundamentally indeterministic setting, and we have given a concrete model of *how* that is possible. We offer our discussion of that concrete model not just as a “how possibly” explanation, but also as a phenomenologically sensible description of a possible mechanism of an associative decision process that may shed light on human or animal agency. Let us hasten to add that we do not claim hereby to have deduced the inner workings of the human brain (that is an empirical task that we leave to the experts), nor to have explained libertarian human free will in all of its complexity and moral significance. We do claim, however, to have opened up a significant route towards understanding free will as based on attributable agency. Our world is indeterministic, and if we are agents, we are agents on that natural basis. Understanding this in detail is certainly hard—but it is not a mystery. Given a concrete model for attributable agency under indeterminism, a blanket rejection of libertarianism is no longer a serious option.

## Footnotes

1 Since the terms “action” and “agency” are used in many different ways, we use the explicit phrase “attributable agency” to stress that our topic is agency that can be attributed to an agent *as an agent*. This requires more than the mere causal involvement of the agent’s body. Importantly, not all physical movements of an agent’s body are attributable in the relevant sense; attribution requires, in some sense that needs to be specified, that the agent *makes a difference*. See our dicsussion in Section “The Agency Dilemma”.

2 A world could be indeterministic in the sense just introduced, and yet such that given *s* at $t_s$, only *e* could happen at $t_e$—such a world would be locally deterministic with respect to $t_s$ and $t_e$. We will ignore this complication in what follows, and take indeterminism to imply different possibilities at $t_e$, given *s* at $t_s$.

3 For a similar assessment, see Steward (2012).

4 See, e.g., Fischer and Ravizza (1998, 253f.), where the authors stress that the truth or falsity of determinism is a scientific issue and argue that while their position is compatible with determinism, it is also consistent with some forms of indeterminism.

5 The discussion given by Fischer and Ravizza (1998, 253) is telling: “[...] the conditions on taking responsibility [...] appear to be straightforwardly consistent with the truth of *certain sorts of indeterminism*. Now this is not to say that these conditions are consistent with *all* kinds of indeterminism. The conditions would not seem to be satisfied in a world in which there is a significant kind of *randomness* at the macroscopic level. [...] But, of course, this is no objection to our theory, as everyone would, presumably, deny that an individual has *control* of the sort required for moral responsibility in a random world.” Never mind the focus on moral responsibility in this quote. The idea seems to be that if an agent’s action is *really* undetermined, then we cannot attribute it any more. But this is then no longer an agnostic position—it relies on the false assumption that all randomness is restricted to a micro-level. Our indeterministic world is not like that.

6 See, e.g., Franklin (2011) for a critical discussion of different arguments and for many references.

7 As in flipping a coin to decide whether to have coffee or tea for breakfast. Note that we would describe such involvement of randomness as part of the agent’s identity.

8 A *contrastive explanation* for the occurrence of an event *e* is an explanation for why *e**in contrast to another event* took place. In an indeterministic scenario, there can be no contrastive explanation in that sense. There can, however, very well be another kind of explanation for the occurrence of *e*. For example, assume that it was genuinely indeterministic whether I would take vanilla or chocolate ice-cream for desert, and I took vanilla. Then, by assumption, there is no full contrastive explanation of why I took vanilla, but there *is* an explanation, I had a reason to take vanilla: e.g., I like vanilla. (There would also have been a reason to take cholocate, that would not have been erratic either: I also like chocolate.)

9 To clarify: we are not saying that there could not be agents that react deterministically in certain types of situations, i.e., agents that always show behavior $b_i$ in situations of type $s_j$. What we are claiming is that the metaphysical assumption of universal determinism is false. (We also believe that given that assumption, we can make no sense of attributable agency.) Our model of attributable agency under indeterminism (see Section “Projective Simulation: A Model for Attributable Agency”), on the other hand, does leave room for learning agents to form deterministic behavior patterns, and can explain these via the development of an agent’s (learning) history. Maybe to be a morally good person means to react deterministically (to choose the one good option) in a wide range of morally challenging situations. We are not claiming that such behavior is impossible, or even strange—we only claim that such behavior, too, needs to be understood on the basis of underlying indeterminism leaving room for learning and development.

10 Libertarianism—the theory that there is free will only in an indeterministic setting—is often held to rely on the following argument: (a) It can be established on conceptual grounds that free will is incompatible with determinism; (b) free will exists; therefore (c) determinism is false. Our dialectics is different. We take both the existence of free will and the incompatibility of free will and determinism as defeasible starting points for investigation. Our main claim is that we have excellent reasons for a belief in indeterminism. This fact *leaves room, but by no means already establishes* a useful notion of free will, or at least of attributable agency, under indeterminism. Our aim is to describe a way in which this room could be filled.

11 See the overview article by Brembs (2011), who cites, e.g., the escape behavior of cockroaches (Domenici et al. 2008).

12 Of course, to decide this question is a task for physics and biology, not for philosophy. For some relevant case studies, see Domenici et al. (2011).

13 This so-called BDI (belief-desire-intention) model for agency, which is very widespread in AI, fits nicely with the causal theory of action proposed by Davidson (1963), which is the mainstream theory of action in philosophy nowadays.

14 Over the years, several cryptographic systems based on pseudo-random number generators have been broken, exposing their insecurity. For one prominent example, see the U.S. National Institute of Standards and Technology’s recommendation to discontinue use of the Dual_EC_DRBG pseudo-random number generator. See http://www.nist.gov/itl/csd/sp800-90-042114.cfm.

15 During a web search conducted on 25 February 2015 we found several online casinos and casino software vendors advertising, e.g., the use of Swiss-based Quantique’s quantum random number generators. See http://www.idquantique.com/ for details.

16 It should be clear that the notion of “option” as described is of course a very primitive one. Since the model of PS does not assume any form of consciousness, the agent will not make any conscious choice. Here, “option” rather refers to the very existence of alternatives, within ECM, which the agent can explore (and possibly evaluate) via simulation, before it takes (i.e., runs into) real action.

17 It is, however, an interesting question, how one would design a “Turing test” that allows, in principle, an external observer to distinguish PS agents based on pseudo-randomness from PS agents based on true randomness.

18 As laid out above, an agent may arrive at deterministic (0 or 1) transition probabilities in its ECM, based on some specific learning history. This is a limiting case in which the challenge of the replay argument vanishes automatically. Here, however, we have to formulate a general reply that also works for non-extremal transition probabilities.

19 As noted in Section “The Agency Dilemma”, this does not mean that the agent’s body wasn’t causally involved in bringing about *e*; that may well be so. What is crucial is that as described, $\alpha$ does not control what happens. In the terminology of Steward (2012), we may say that attribution is impossible because the agent is treated as *being a body*, rather than as *having a body*.

20 Since there are well-defined transition probabilities for these steps, we can cite these probabilities to underline to what extent the actual step was possible. But note that even in case that probability is very low, the step can just occur. It is a mistake to think that quoting a probability can give a contrastive explanation of why one rather than another step occurred.

21 We do not think that an agent’s integrity would be threatened if, say, its choice for vanilla versus chocolate ice cream in a given situation is completely random. Each such choice is still the agent’s, and via external feedback will contribute to its development. Attributability stays intact, no matter whether an explicit use of external randomness is involved (flipping a coin) or the choice is due to the indeterministic constitution of the agent.
